# Digital-based physical activity interventions implemented across the League of Arab States: a scoping review

**DOI:** 10.1093/oodh/oqaf028

**Published:** 2025-11-03

**Authors:** Elizabeth Dodge, James Barry, Fatmah Almoayad, Samiah Alqabbani, Afrah Almuwais, Joanna Kruk, Basil H Aboul-Enein

**Affiliations:** Applied Nutrition Graduate Program, University of New England, College of Professional Studies, 716 Stevens Ave. Portland, ME 04103, United States; Physical Education Teacher Education, East Stroudsburg University, 200 Prospect Street, East Stroudsburg, PA 18301, United States; Department of Health Sciences, College of Health and Rehabilitation Sciences, Princess Nourah Bint Abdulrahman University, Airport Road, Riyadh, Saudi Arabia; Department of Rehabilitation Science, College of Health and Rehabilitation Sciences, Princess Nourah Bint Abdulrahman University, Airport Road, Riyadh, Saudi Arabia; Department of Rehabilitation Science, College of Health and Rehabilitation Sciences, Princess Nourah Bint Abdulrahman University, Airport Road, Riyadh, Saudi Arabia; Faculty of Physical Culture and Health, University of Szczecin, Al. Piastów 40b/6, Szczecin 71-065, West Pomeranian Voivodeship, Poland; College of Arts & Sciences Health & Society Program, University of Massachusetts Dartmouth, 285 Old Westport Rd, North Dartmouth, MA 02747, United States; Faculty of Public Health and Policy, London School of Hygiene & Tropical Medicine, 15-17 Tavistock Place, London WC1H 9SH, United Kingdom

**Keywords:** physical activity, digital health, middle east, north Africa

## Abstract

This scoping review investigates the use and impact of digital-based physical activity (PA) interventions in Arabic-speaking countries. These technologies have dramatically transformed healthcare management and offer a valuable tool for managing non-communicable diseases. Despite challenges such as limited healthcare access and cultural norms, these tools enable health promotion, preventive care, and personalized health plans. The aim of this scoping review is to evaluate the effectiveness of digital-based PA interventions, such as mobile apps, used across the League of Arab States. PRISMA-ScR guidelines were applied to conduct this scoping review across 10 databases using pertinent search terms for relevant studies published between 2010 and December 2024 to identify publications conducted across Arab countries. Sixteen studies on digital-based PA interventions in four Arab countries were analyzed. The most effective interventions blended various digital and educational strategies, leading to significant increases in PA levels and associated anthropometric outcomes. Despite promising results, there was no evidence that the interventions’ effects were sustained over the long term, and the studies’ geographical coverage was limited, emphasizing the need for larger-scale, diverse studies to assess long-term effectiveness of digital-based PA interventions.

## INTRODUCTION

### Digital-based interventions for non-communicable disease

Non-communicable diseases (NCDs), including cardiovascular disease (CVD), type II diabetes mellitus, cancer, and musculoskeletal disorders, are the leading causes globally, of morbidity and mortality [1–3]. According to the World Health Organization, over 41 million people die each year from NCDs, accounting for roughly 74% of all deaths worldwide [4]. The burden is disproportionately borne by people of lower socioeconomic status, those without access to consistent healthcare, and minority populations in countries with advanced economies [5–9], while also having a disproportionate impact on low-and middle-income-countries (LMIC’s) [3, 5, 10]. Across the Organization for Economic Cooperation and Development countries, low socio-economic status is consistently and significantly associated with NCD related morbidity and mortality [8]. Digital-based and digitally delivered interventions can provide low-cost, easily accessible interventions that target behavior change related to non-communicable diseases, and have been shown to be effective at promoting positive health behaviors to help prevent or manage NCD’s [11–13]. Interventions target a range of behaviors associated with NCD’s, including, but not limited to, mental well-being, smoking cessation, adoption of healthy eating behaviors, and increasing physical activity (PA) [14–17].

### Digital-based PA interventions

One of the key benefits of digital- and app-based interventions is their ability to empower individuals to take control of their health and wellness [18]. These technologies promote health lifestyles by providing users with real-time feedback on their health metrics, such as PA, heart rate, sleep patterns, and more, enabling them to make informed decisions about their lifestyle and healthcare management [19]. Digital-based interventions specifically focused on increasing PA have been shown to be effective to varying degrees when supplied by various digital and mobile delivery modalities (email, text message, zoom, WhatsApp) as well as across varied populations; the evidence shows a consistent positive, yet sometimes weak association between digitally delivered interventions and their outcomes [20–24].

### Digital-based interventions in the Arab States

One of the most prominent health challenges in Arabic-speaking countries is the increasing prevalence of non-communicable diseases (NCDs) such as cardiovascular diseases, diabetes, and obesity. Risk factors for NCDs, such as unhealthy diet, physical inactivity, and sedentary lifestyles, are prevalent in the region, contributing to the increasing burden of NCDs. Lifestyle changes associated with urbanization and economic development have also contributed to the rising prevalence of NCDs in Arabic-speaking countries [25–29]. A recent review found that digital health interventions in fragile states in the Middle East and North Africa can provide solutions to help address health needs in areas of geopolitical instability [30]. A digital health intervention designed to help displaced persons with depression and/or anxiety found clinically relevant improvements in participants that had consistent digital access’ noting that the success of such interventions is limited by access to electricity and technology with which to engage with them [31]. A review by Al Dweik et al [32] found that digital-based mental health interventions implemented in the United Arab Emirates were feasible, accessible, flexible, and often improved clinical outcomes; however challenges such as participant level of digital literacy, access to technology, reading ability, and concerns that digital modality could totally replace (to the detriment of the client) face-to-face or physical interactions with their mental health professionals [32]. The League of Arab States includes both low-and middle-income countries, as well as those that have more advanced economies; while this review predominantly includes studies that are not conducted in LMIC’s, there are shared cultural norms, health beliefs, and similar social and political structures to navigate in the provision of public health interventions. Therefore, it is critical to examine potential challenges and barriers to the delivery of digital-based health interventions in the Arab States, while also identifying commonalities in successful digital-based interventions that can be applied to increase efficacy of such interventions.

### Digital-based PA interventions in the Arab States

The use of mobile health app technology has been used to encourage the population in Arabic-speaking countries to follow PA guidelines which is essential for improving overall health and well-being. Implementing health promotion interventions, programs, and campaigns targeting different communities, such as schools, workspaces, and public places, can be effective in promoting and maintaining PA [33–38]. Mobile health promotion interventions focused on developing strategies that foster individual motivation to start walking regularly and maintain their commitment to the activity. Identifying and addressing barriers to walking, such as time constraints, lack of motivation, or safety concerns, can contribute to enhancing individual commitment to walking as a regular PA [39–43]. Culturally relevant educational campaigns that integrate the health benefits of exercise into familiar cultural narratives can increase intervention adherence and efficacy, including culturally tailored interventions, community-based initiatives offering gender-specific exercise classes, walking groups, or fitness events within culturally safe environments. Addressing these challenges is critical to fostering equitable opportunities for PA [44].

The advent of wearable and digital health technology and the use of health apps on mobile phones have revolutionized the way individuals manage their health and wellness, gaining popularity worldwide, including in Arabic-speaking countries, and are transforming the landscape of healthcare management [45]. Advancements in digital-based technology have enabled the integration of digital devices, mobile applications, and communication tools, such as activity monitors and digital mobile applications, to influence behavior positively and encourage the adoption of a healthier lifestyle [46]. In Arabic-speaking countries, where access to healthcare services may be limited in certain areas or for certain populations, wearable health technology and health apps can bridge the gap by allowing remote monitoring of patients’ health metrics. Another significant advantage of wearable health technology and mobile technology health apps is their potential to enhance remote monitoring and telehealth, patients can wear monitoring devices that record vital information that can get uploaded remotely to healthcare providers [43].

The state of health in Arabic-speaking countries is shaped by a range of factors, encompassing social, economic, environmental, and cultural determinants of health [44, 47, 48]. According to the World Health Organization Eastern Mediterranean Regional Office, the rate of non-communicable diseases associated with obesity-related comorbidities such as diabetes and heart disease is rapidly rising in this region [25, 44, 49–51]. Therefore, this scoping review aims to evaluate the effectiveness of digital-based PA interventions, such as mobile apps, used within the League of Arab States. This information is essential for suggesting culturally and socially appropriate health promotion strategies that address sedentary lifestyle behaviors in Arabic-speaking communities.

## METHODS

The scoping review was performed in accordance with the PRISMA extension for scoping reviews and Arksey and O’Malley’s framework [52, 53]. The search was conducted in the winter of 2024 and the results communicate literature published between 2010 and December 2024. For the purpose of this review, Arabic-speaking countries are defined as the 22 member countries of the Arab League States [54, 55]. Ten academic electronic databases were used for the literature search: Scopus, PubMed, SpringerLink, Wiley Online, ProQuest, ArticleFirst, EBSCOhost, Taylor & Francis, Web of Science, and ScienceDirect. These databases were selected due to their medical and biomedical scope. The following combination of search terms, keywords, and phrases were used: ‘Online’; ‘app’; ‘digital’; ‘computer’; ‘technology’; ‘internet’; ‘web-based’; ‘social media’; ‘eHealth’; ‘mHealth’; ‘smartphone’; AND ‘Physical Activity’; ‘Exercise’; ‘Intervention’; ‘Program’ AND ‘Algeria’; ‘Egypt’; ‘Bahrain’; ‘Comoros’; ‘Djibouti’; ‘Iraq’; ‘Jordan’; ‘Saudi Arabia’; ‘Kuwait’; ‘Lebanon’; ‘Libya’; ‘Mauritania’; ‘Morocco’; ‘Oman’; ‘Occupied Palestinian Territories’; ‘Qatar’; ‘Yemen’; ‘Somalia’; ‘Sudan’; ‘Syria’; ‘Tunisia’; ‘the United Arab Emirates’ (See supplemental material file and Table 1).

**Table 1:** Electronic databases used with relevant search period and terms

Databases	Search Period	keywords, search terms, and phrases
Scopus, PubMed, SpringerLink, Wiley Online, ProQuest, ArticleFirst, EBSCOhost, Taylor & Francis, Web of Science, and ScienceDirect	2010 and December 31^st^, 2024	‘online’ [All Fields]; OR ‘app’ [All Fields]; OR ‘digital’ [All Fields]; OR ‘computer’ [All Fields]; OR ‘technology’ [All Fields] OR ‘internet’ [All Fields] OR ‘web-based’ [All Fields] OR ‘social media’ [All Fields] OR ‘eHealth’ [All Fields] OR ‘mHealth’ [All Fields] OR ‘smartphone’ [All Fields] AND ‘Physical Activity’ [All Fields]; OR ‘Exercise’ [All Fields]; AND ‘Intervention’ [All Fields]; OR ‘Program’ [All Fields] AND ‘Algeria’; ‘Egypt’; ‘Bahrain’; ‘Comoros’; ‘Djibouti’; ‘Iraq’; ‘Jordan’; ‘Saudi Arabia’; ‘Kuwait’; ‘Lebanon’; ‘Libya’; ‘Mauritania’; ‘Morocco’; ‘Oman’; ‘Occupied Palestinian Territories’; ‘Qatar’; ‘Yemen’; ‘Somalia’; ‘Sudan’; ‘Syria’; ‘Tunisia’; ‘the United Arab Emirates’ [All Fields]

**Figure 1:**
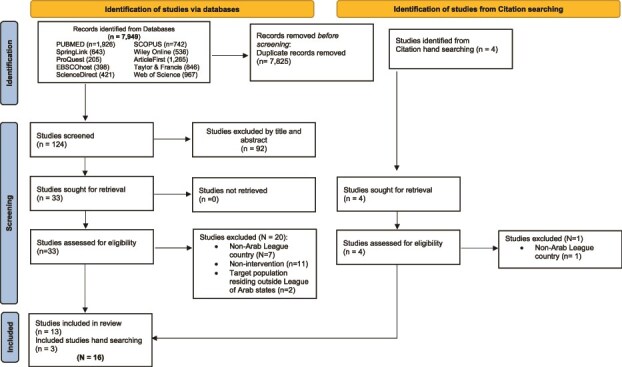
PRISMA 2020 flow diagram

The search strategy was customized to fit the indexing systems of each respective database. All retrieved articles were screened for their relevance to the topic. Additionally, reference lists of included studies were examined for further relevant studies (see Fig. 1). Titles and abstracts were assessed for relevance, and pertinent journal abstracts were reviewed by three authors (J.K., E.D., and B.A-E.). One author (B.A-E) utilized Rayyan QCRI software to aid in the screening process [56]. Potential disagreements between reviewers were resolved by consensus. Potential studies for inclusion were independently evaluated based on relevance, merit, and eligibility criteria for inclusion and exclusion (see Table 2). Final decisions regarding inclusion and the documentation of exclusion reasons were made collectively by all authors. Figure 1 illustrates the elimination process leading to the selection of articles for this review. The data extracted from the studies included in the review include author, year of publication, country of intervention, the target population, sample size, intervention type, details of the intervention as related to digital-based delivery of an intervention measuring PA and related anthropometrics as outcomes, the intervention duration, the theoretical framework or model used in intervention development, the outcome measures, main findings, and main recommendations. The characteristics of the data extracted from the included studies are summarized in Table 3. Methodological quality assessment is not a requirement for scoping reviews, included studies were not evaluated for quality [57]. No ethical oversight was found to be necessary for this review and, therefore, no institutional review board was acquired.

**Table 2:** PICOS criteria for inclusion and exclusion of studies

Parameter	Inclusion Criteria	Exclusion Criteria
Population	Arabic speaking populations that reside in an Arab League member state	Non-Arab or Arabic speaking populations Arab diaspora residing outside an Arab League member state.
Intervention type	Any kind of digital based physical activity (PA) intervention that address PA-related outcomes, including: Digital Applications of PA Digital Tools in enhance PA Educational interventions. Environmental Interventions.	Non-digital based Interventions Interventions that do not address PA-related outcomes.
Comparators	Pre-intervention, baseline physical activity-related variables (i.e. anthropometric measures, PA-related knowledge, self-reported scales related to PA) of groups who were: Control: received no intervention. Received partial intervention (e.g. educational intervention only vs. multi-componential intervention.)	N/A
Outcomes of interest	Changes in anthropometric outcomes, e.g.: BMI, WC, weight Changes in self-reported scales related to PA outcomes. Changes in PA-related knowledge. Changes in meeting the PA daily recommendations. Changes in adherence to PA.	Non-PA related outcomes
Language	English, Arabic, French	All other languages
Study Type	Experimental intervention studies with quantitative outcomes. Peer-reviewed original research articles Original research conference publications	Non-numeric/categorical assessments or qualitative studies Non-Peer-reviewed articles Study protocols Narratives Similar article types Grey literature Communications Non-intervention based studies White papers

Abbreviations

Pa- physical activity

BMI – Body Mass Index

N/A – Not applicable

WC: Waist Circumference

**Table 3:** Digital-based physical activity interventions in Arabic-speaking countries (N = 16)

Intervention Author (year)	Country	Target Population	Type of Study	Sample Size	Intervention Type(s)	Details of PA Intervention	Duration of Intervention	Theoretical Framework/ Model used	Measured Parameters related to PA	Main Results	Main Recommendations
Phone Calls											
Gmmash et al. (2023).	Saudi Arabia	Adolescents (10–18 yo)	Pre- and post-experimental study	27 participants	Phone Calls Virtual (zoom) training on PA	As part of the intervention group participants received a motivational phone call from a trained physical therapist.	8 weeks	Self-Determination Theory	Physical Activity Level The Godin Leisure time Exercise Questionnaire Situational Motivational Scale	Participants had significant improvement on a PA scale (*P =* 0*.*13) and significant changes in situational motivation scale (*P =* 0*.*42). These improvements were across groups, suggesting there is no additional benefit to the phone calls in addition to the virtual training.	Significant improvements were detected in both groups after the virtual eight-week program. Preliminary data suggest that providing a virtual physical education program can improve adolescents’ physical health.
Mobile App											
Shaban et al., (2024).	Egypt	Adults with type II diabetes, over 50	Quasi-experimental pretest-post-posttest design	120	Mobile app	The intervention group received access to the app, which provided personalized content on multiple factors, including PA. The control group received SOC and face-to-face counseling, with print educational materials	4 months	Social Cognitive Theory, Self-efficacy	Summary of Diabetes Self-Care Activities (SDSCA), specifically the exercise section	The post- intervention group score on the exercise portion of the SDSCA statistically significantly increased (*P* < 0.001) compared to the control	This study demonstrates the possibility of a digital-based nursing intervention to improve self-care behaviors in adults with Type II Diabetes Mellitus, including increasing exercise
Basuodan et al. (2023).	Saudi Arabia	Female Junior College students aged 19 (+/−0.9) years	Pre-posttest design	46	Virtual Introductory (zoom) class WhatsApp	6-week course where students received daily online PA promotive messages via the WhatsApp smartphone application. One message every working day (5x/week)	6 weeks	Social Cognitive Theory, Self-efficacy	physical activity IPAQ-Long form, and IPAQ-short form Metabolic Equivalency (MET)	Significant increase in the proportion of students who perform walking (*P =* 0*.*2) and in moderate PA (*P =* 0*.*2) for leisure. No significant changes in any other area	The program is effective in increasing college students PA in specific domains such as walking for transportation and engaging in moderate PA for leisure, but not in improving overall PA levels.
Alshahrani et al. (2021).	Saudi Arabia	Female College Students Age 18–28	Pretest/posttest Randomized controlled open label experimental design	110	WhatsApp	Using WhatsApp, the intervention group received a 15-minute orientation and 3 to 4 health-promotional/physical activity messages per week.	10 weeks	Health Belief Model of Behavior Change	Baseline physical activity between control and intervention group Baseline and postintervention physical activity in control and intervention groups. MET	Significant difference was observed in the intervention group pre and post in all domains (work-related, (*P* < 0.001), transportation related, (*P* < 0.001), recreational (*P* < 0.006), and total PA, (*P* < 0.001),	Technological advancements can be used as a tool for health promotion.
Ismail et al. (2022).	Qatar	Employees in Qatar who spend most of the working day sedentary.	Mixed Methods	58 participants	Mobile App	Two apps were used MotiFitLite which sent a static message to the control group Moti Fit sent a context aware personalized message to promote PA	66 days	Health Belief Model of Behavior Change	Physical Activity time	Neither group showed a significant monotonic trend in daily active time. The intervention group had a higher level of engagement (*P* < 0.001) and increased PA at work (*P* < 0.001) compared to the group receiving static messages, however, there was not a significant increase in overall activity (*P =* 0*.*6) compared to the control.	The Motifit lite encouraged users to take a break but did not show a significant difference in PA. A larger sample size could potentially show stronger results.
Alasfour, & Almarwani (2022).	Saudi Arabia	Women aged >50 with knee osteoarthritis (OA)	Two-arm RCT parallel study design	40	Smartphone App	‘My Dear Knee’ is an Arabic Smartphone Application	6 weeks	n/a	Self-reported exercise adherence	My Dear knee intervention group had statistically significantly higher adherence to exercise (*P =* 0*.*02)	The positive results on adherence rate suggest that this simple intervention is potentially effective. The use of remote technology appears to overcome some barriers that may limit older Saudi women from receiving supervised physical therapy
Ali et al. (2021).	United Arab Emirates	Women with overweight or obesity	Non-randomized, two-arm feasibility study	161	Self-Monitoring Fitness App	Rashakaty website Self-Monitoring tools in the MyNetDiary commercial app	16 Weeks	Social Cognitive Theory	Physical Activity Levels WC BMI % body fat Amt body fat	Compared to the basic intervention arm, the enhanced intervention arm demonstrated significant increases in minutes of vigorous PA (*P =* 0*.*25), days of moderate PA (*P* < 0.001), minutes of moderate physical activity (*P* < 0.001), and walking (*P* < 0.001), as well as a significant decrease in sitting time (*P* < 0.001). Anthropometrics related to PA also exhibited change in the enhanced intervention arm compared to the basic, with significant decreases in WC (*P* < 0.001), BMI (*P* < 0.042), % body fat, (*P* < 0.001), amount boy fat (*P* < 0.001), with a decrease in fat-free mass (*P =* 0*.*43) compared to the basic intervention group.	Use of language on website and educational materials should be more inclusive and culturally tailored. Mobile-and technology based education delivery can improve PA, and associated anthropometrics.
Alnasser et al. (2019).	Saudi Arabia	Women with overweight or obesity	Pre-test/post-test single arm	240	Phone App	Evidence Informed Mobile Health app (Twazon Arabic weight loss app)	4 months	Health Belief Model of Behavior Change	Physical Activity Weight WC	Limited differences were noted between engaged and unengaged app users; WC decreased significantly (*P* < 0.01) compared to unengaged users, and while not significant (*P =* 0*.*18), engaged users experiences greater weight loss than ungagged users. PA was not significantly different between groups (*P =* 0*.*49).	Positive outcomes were seen but the limited sample size did not allow for any concrete conclusions.
Pedometer											
Hasan et al. (2018).	United Arab Emirates	Adult females	Quasi-experimental pretest-posttest	52	Pedometer	Each participant was given a digital pedometer to wear and a physical activity logbook. Weekly follow-up was conducted to ensure progress.	9 weeks	Lifestyle modification based educational material	PA Step Count Anthropometric Assessments	There was a noticeable but not significant increase in steps. However, a significant reduction in all measured anthropometric parameters was found, with the exception of fat free mass.	The findings suggest that participants in both the low active and high active lifestyle groups benefited from counting steps using a pedometer.
Multi-modality											
Saquib et al. (2023).	Saudi Arabia	Female	2 * 2 randomized trial design	181	WhatsApp Pedometer	Participants in the intervention group used a WhatsApp group chat, received two to three health promotional WhatsApp messages, and given pedometers for step count. Control group receives similar number of WhatsApp non-health related messages and no pedometer.	12-Week	Social Cognitive Theory	Pedometer Step Count Self-reported average number of minutes per day was calculated using IPAQ	There was no significant difference between group step counts; however, the intervention group had a significant group-by-time change in average daily step count (*P =* 0*.*4) compared with the control group.	Delivering a PA intervention to university students through WhatsApp is a feasible and potentially effective way to increase daily steps
Al-Kuwari et al. (2017).	Qatar	Adults in Qatar	Longitudinal Intervention	268	Pedometer Emails and Text Messages	Pedometer and an online self-monitoring online account	1 year	Social Cognitive Theory	Physical Activity (Steps per Day)	Significant increase in steps per day (*P* < 0.05)	Pedometer based intervention has long term effects on increasing physical activity levels
Al-Ghafri et al. (2021).	Oman	Adults aged 18–60 with type 2 diabetes and no contraindications to Physical Activity	Quasi-experimental pretest-posttest	174	WhatsApp Pedometer	Each member in the intervention group received a PA consultation, Pedometer, and standardized messages using WhatsApp Messenger App	12 months	Health Belief Model, Stages of Change, and Social Cognitive Theory	Physical Activity using 13-item Global Physical Activity Questionnaire (self-efficacy SE, social support SS) and expressed as MET equivalent	The intervention group scored higher in self-efficacy in engaging with PA (*P* < 0.001), following PA instructions, (*P <* 0*.*01), and managing time to engage in PA (*P <* 0*.*01).	The MOVEdiabetes intervention for physical activity was associated with positive changes in self-efficacy related PA.
Al-Mohannadi et al. (2019).	Qatar	Health Care Workers in Qatar	Two Cross Sectional Surveys, pre-post intervention	212	Pedometers Email tips Text messages	Step into Health Participants received a pedometer that uploads to an online platform The 3-month workplace challenge included health tip sent via email and text message Participants who averaged 10 000 steps per day were randomly selected to receive incentives	3 months	Social Cognitive Theory	Step Count International Physical Activity Questionnaire (IPAQ)	Average step count was significantly increased from pre-post intervention (*P =* 0*.*48), while males in the study were significantly more active (*P =* 0*.*24).	Step Into Health workplace interventions showed an increase in PA, but those numbers decreased after the intervention period.
Al-Kuwari et al. (2016).	Qatar	Adults	Cross-sectional longitudinal study	970 Adults	Pedometer Email Text messages	Email reminders when participants did not upload data for 14, 21, and 28 days	12 weeks	Social Cognitive Theory	Step count	There was a significant increase in daily step count at 12-weeks (*P =* 0*.*01)	Pedometer programs combined with email and text reminders can be used to promote physical activity among middle-aged adults and elderly to prevent non-communicable diseases associated with sedentary lifestyles
Al-Kuwari & Al-Hamdani (2021).	Qatar	Male participants Age from 18 – more than 60	Retrospective study	1127 Participants	Pedometer and mobile app	518 used pedometer and 609 used a smartphone app	12 weeks	Social Cognitive Theory	Step Count	Both groups showed significant changes from pre-post intervention. The pedometer group significantly increased their daily steps (*P =* 0*.*01), while the smartphone app group also exhibited significantly increased steps from pre-post (*P <* 0*.*5). There was no significant difference in outcome between the two groups (*P =* 0*.*61)	The findings suggest that the use of the Step Into Health program when combined with either a programmed digital pedometer, or the use of a smartphone pedometer app can significantly increase PA.
Salih Khidir et al. (2021).	Qatar	University Staff and Students ages 18–64	Experimental pretest-posttest design	N = 397 n = 288 phase 1 n = 109 phase 2	Pedometers or mobile app Initial presentation on PA, access to Steps in Health website	All participants were invited to take part in a 4-month walking competition between participating campuses.	5-months phase 1- and 5-months phase 2	Social Cognitive Theory	Step Counts	Significant increase in step uploads during phase 1 where participants received reminders, however there was not a significant increase in steps in either phase.	The study showed a mix of walking competitions, and use of mobile technology to evaluate step counts did not statistically significantly increase steps, however, the reminders to upload daily step activity were effective.

Abbreviations; Bmi: body mass index; MET: Metabolic Equivalency; PA: physical activity; SOC: Standard of care; WC: Waist Circumference

## RESULTS

This review included 16 studies that were conducted between 2016 to 2024, in five Arabic-speaking countries. Six studies were conducted in Saudi Arabia [58–63]. Six studies were also conducted in Qatar [64–69]. Two studies were conducted in The United Arab Emirates [70, 71] and a single study was conducted in each Oman [72] and Egypt [73]. The research designs employed in these studies were diverse, and included retrospective [57], cross-sectional [56, 58], randomized controlled trials [47, 49], and pre-post experimental designs [48, 50, 51, 59, 62, 63], among others described in Table 3.

The studies included in this review spanned a wide range of sample sizes, and included a total of 4183 participants. The largest sample size was in the retrospective study conducted by Al-Kuwari & Al-Hamdani [66] and included 1127 participants while the smallest sample size was 27 participants in a pre-post–test experimental pilot study [62]. The duration of the included interventions ranged from 6 weeks [58, 61], and 1 year [69, 72], with a mean intervention duration of 16.77 weeks. The participant age also ranged greatly, from Adolescents 10–18 [62] to adults aged 64 [68], indicating that digitally delivered interventions use may be an effective tool to engage with a variety of ages.

Of the 16 studies included in this review, 15 explicitly incorporated a theoretical framework or model into their methodologies. The Self-Determination Theory was applied in the study by Gmmash et al [62]. The most commonly employed theoretical basis for the interventions was the Social Cognitive Theory (SCT) which was utilized in 10 interventions [61, 63, 65–70, 72, 73], with Basuodan et al [61], and Shaban et al [73] both using SCT and emphasizing the construct of self-efficacy [50] and Al-Ghafri et al [72] using SCT in combination with the Health Belief and Stages of Change models [63] . Other interventions relied on the Health Belief Model of Behavior Change [59, 60, 64], or Lifestyle Modification [71] as the framework to elicit behavior change. Only one included study by Alasfour & Almarwani [58] did not explicitly note a theoretical framework or model that the intervention was based upon; the ‘My Dear Knee’ app was designed to help women with knee osteoarthritis complete PT exercises.

The 16 studies included in this review employed a diverse range of interventions, leveraging various digital tools and strategies. The majority of the interventions included utilized an app to deliver the intervention that relied solely on an app for delivery [58–61, 64, 70, 73], while others [63, 65–69, 72] used a multi-modality approach that combined apps, emails, and/or text messaging with pedometers. Hasan et al [71] used just a digitally programmed pedometer and a PA logbook to track their steps in. Finally, Gmmash et al [62] utilized zoom to train participants on PA, followed by weekly phone calls from a trained physical therapist. The parameters of interest extracted from each study were the outcome measures related to PA, as well as related anthropometric changes.

The majority of the studies reported a significant impact of the digitally delivered intervention on PA, or related anthropometrics. Five studies utilizing a PA scale to measure activity saw statistically significant increases in PA score [61, 62, 65, 72, 73] in the intervention groups, while Saquib et al [63] reported no between group differences, however, they did find a group-by-time change (*P =* 0*.*4) in the intervention groups daily step count.

In studies that reported PA as step count, there were mixed findings. Hasan et al [71] found a noticeable but non-significant increase in steps, however interestingly, they did find statistically significant improvements (significance was set at *P <* 0*.*5) across all anthropometric measures with the exception of fat-free-mass; these included improvements in BMI, Body fat mass, waist:hip ratio, waist circumference, and visceral fat area. These findings indicate that even a nominal increase in steps can have positive and significant impacts on indices of obesity. However, three studies [65, 67, 69] demonstrated that their digital-based intervention significantly increased step count in the intervention group (*P* < 0.05, *P =* 0*.*48, *P =* 0*.*01, respectively), while one study [66] found that both their pedometer group and their smartphone app group significantly increased their step count from pre-post intervention (*P =* 0*.*01, and *P* < 0.05, respectively), there was no significant difference between groups (*P =* 0*.*61), indicating that the intervention + either a digital pedometer or a smartphone app to track steps can contribute to increased step counts. Salih Khidir et al [68], did not find any increase in steps between phases of the intervention, however they did note that in phase one when there were participant reminders to log their steps that there was a significant increase in the uploading of steps data, indicating that incorporation of periodic reminders or ‘nudges’ to participants can increase adherence to intervention protocol. Ali et al [70] found statistically significant increases in minutes of vigorous PA (*P =* 0*.*25), days of moderate PA (*P* < 0.001), minutes of moderate PA (*P* < 0.001), and walking (*P* < 0.001), as well as a significant decrease in sitting time (*P* < 0.001) in the enhanced intervention group compared to the basic intervention group. Alshahrani et al [60] reported significant findings in time spent in PA from pre-post in the intervention group across all domains, including time spent in PA at work, related to transportation (i.e. walking, rather than driving, parking further away from your destination), recreational PA and total PA. Also reporting on time spent in PA, Ismail et al [64] found no significant difference between the intervention and control groups in overall activity, however, the intervention group did have significantly increased PA at work. One study [58] investigated self-reported exercise adherence in the ‘My Dear Knee’ app; which found that the intervention group reported significantly higher (*P =* 0*.*02) adherence to exercise compared to the control group. Anthropometrics related to PA also exhibited change in the enhanced intervention arm compared to the basic, with significant decreases in WC (*P* < 0.001), BMI (*P* < 0.042), % body fat, (*P* < 0.001), amount boy fat (*P* < 0.001), with a decrease in fat-free mass (*P =* 0*.*43) compared to the basic intervention group [70].

## DISCUSSION

This scoping review identified 16 studies conducted between 2016 and 2024 that evaluated the impact of digitally delivered PA interventions across five Arabic-speaking countries. The majority of the included studies demonstrated that digital interventions, whether app-based, multi-modal, delivered via phone or complimented using pedometers, were effective in increasing PA levels and improving related anthropometric outcomes. Notably, five studies [61, 62, 65, 72, 73] that measured PA using validated scales reported statistically significant improvements; these five studies all utilized a theoretical framework to inform the development and delivery of the intervention materials, and include Self-Determination Theory [62], Social Cognitive Theory (SCT) with Constructs of Self-Efficacy [61, 73], Health Belief Change combined with Stages of Change and SCT [72], and Social Cognitive theory [65]. While connected but distinct concepts, each of these interventions conceptual frameworks utilized the construct of self-efficacy to effect behavior change.

The findings indicate that digitally delivered interventions can be powerful tools for addressing sedentary behaviors and associated non-communicable diseases (NCDs) in Arabic-speaking populations, and build upon recent literature noting that purposeful use of social media/digital interventions, especially when culturally tailored, can effect behavior change in this population [73–77]. These findings align with the success of digitally delivered public health interventions globally [78–81]. Consistent with prior research, the reviewed interventions delivered through mobile apps and wearable devices provided participants with tools to monitor progress, receive feedback, and sustain engagement over time. The role of self-monitoring and feedback was particularly evident in studies where reminders or ‘nudges’ led to improved adherence [60, 61, 65, 67–69, 72], suggesting that ongoing interaction and support are key components of success. While many of the included interventions demonstrated improvements in PA behaviors, the heterogeneity of study designs and populations (15/16 articles were from Arabic-speaking countries that are considered advanced economies, so adaptations for use in LMIC Arab-Speaking nations are needed, and this is an area for future research) highlights the need for culturally tailored approaches, particularly if these approaches are to be used across the 22 nations within the League of Arab States. For example, interventions that incorporate gender-specific programming, culturally relevant messaging, and considerations for local health beliefs showed higher engagement and effectiveness [82–85]. This reinforces the importance of embedding digital interventions within the social and cultural context of Arabic-speaking countries to enhance uptake and sustainability.

The results of this review align with global literature showing the effectiveness of digital health interventions in promoting PA. Consistent with prior research, interventions delivered through mobile apps and wearable devices provided participants with tools to monitor progress, receive feedback, and sustain engagement over time [86–89]. Interestingly, even interventions resulting in modest increases in steps demonstrated meaningful improvements in anthropometric indicators such as BMI, waist circumference, and body fat percentage, underscoring the potential for small, sustained changes to yield significant health benefits. [71, 86]

Given the rising prevalence of NCDs in the region, these findings have significant implications for public health policy and practice. Digital interventions present a scalable and cost-effective strategy to reach diverse populations, including those in rural or underserved areas with limited access to traditional healthcare services. Importantly, the use of mobile apps and wearable devices provides opportunities for real-time data collection and remote monitoring, which can be integrated into healthcare systems to improve population-level surveillance and management of chronic conditions.

However, the success of these interventions is dependent on addressing barriers such as digital literacy, cultural tailoring and acceptability, access to reliable technology, and user engagement. It is important to recognize that geographical coverage in digital health interventions plays a crucial role in the culturally diverse Arab League, which consists of 22 member countries, each with its own unique cultural, social, and economic contexts. This diversity impacts health behaviors and the adoption of technology. By including various countries helps to ensure that specific population needs are addressed and to enhance the generalizability of research findings [90].

Understanding the necessary cultural and linguistic adaptations is key for the successful implementation of digital health interventions within diverse populations. For instance, adapting digital programs for Syrian refugees in the UK underscores the importance of cultural sensitivity when tackling mental health concerns, such as suicidal thoughts [91]. Language and cultural barriers may hinder individuals’ ability to effectively utilize digital health resources. This issue is also evident in Germany, where Arabic-language digital programs experience lower adoption rates compared to their German counterparts [30]. Ensuring that these interventions are culturally and linguistically tailored can significantly increase their accessibility and effectiveness.

In lower-income Arab countries, the adoption of healthcare technology is affected by inadequate infrastructure, low digital literacy, and inconsistent regulatory frameworks [92]. This is because many of these nations lack the necessary resources and infrastructure to utilize digital health tools effectively. To address these challenges, it is critical to employ strategies such as user-centered design, seamless integration of new tools with existing systems, and gain political support. Economic constraints further complicate this situation, as limited funding restricts investment in essential infrastructure [93]. However, with improved planning and investment, these obstacles can be navigated, ultimately leading to enhanced healthcare access and outcomes across the region.

The increasing utilization of mobile phones in low- and middle-income countries enhances the ability of digital health services to reach a broader audience across the league of Arab States, especially in resource-limited areas. This allows telemedicine and digital technologies to significantly improve healthcare delivery by facilitating disease diagnosis, enabling early illness detection, reducing costs, and providing essential health services in a more accessible manner, particularly for individuals in remote or underserved regions [94].

The finding that reminders and behavioral prompts enhanced adherence suggests that interventions should prioritize interactive features that foster motivation and accountability. Furthermore, collaboration between public health officials, technology developers, and community stakeholders will be essential to design solutions that are equitable and inclusive.

### Strengths and limitations

This review highlights the growing body of research supporting the use of digital PA interventions in Arabic-speaking countries. A notable strength of the included studies was the widespread integration of behavior change theories, such as SCT and the Health Belief Model, providing a strong conceptual foundation for intervention design. The diversity of study designs, ranging from randomized controlled trials to pre-post experimental studies, also offered a comprehensive view of intervention effectiveness.

Nevertheless, several limitations warrant consideration. The variability in intervention duration, delivery modality, and outcome measures made direct comparisons challenging. Some studies had small sample sizes, which may limit generalizability. Additionally, only one study was conducted in low- and middle-income countries across the League of Arab States, despite the significant burden of NCDs in these countries. This gap highlights a need for future research that prioritizes equity and accessibility across the full range of Arabic-speaking populations within this region.

### Future directions for research

Future studies should focus on long-term sustainability and scalability of digital interventions. Research exploring strategies to integrate digital health tools into existing healthcare systems will be critical, particularly for chronic disease management. Additionally, more rigorous designs, including large-scale randomized controlled trials, are needed to strengthen the evidence base. Efforts should also be directed toward understanding how gender, socioeconomic status, and cultural norms influence engagement and outcomes, to ensure interventions are both effective and culturally sensitive.

## CONCLUSION

Digital health interventions represent a promising avenue for increasing PA and addressing the growing burden of NCDs in Arabic-speaking countries. This review demonstrates that such interventions can effectively improve PA behaviors and related health outcomes when thoughtfully designed and culturally adapted. By leveraging technology and behavior change theory, there is an opportunity to create sustainable, accessible solutions that promote health equity and support population-level wellness. The evidence from this review suggests a promising potential for digital-based PA interventions in enhancing the health status and behaviors of populations. A range of interventions, encompassing diverse strategies such as phone calls, mobile applications, social media platforms, and wearable devices like pedometers, yielded various degrees of success, with several studies demonstrating significant improvements in PA levels, anthropometric measurements, dietary habits, and psychosocial parameters. Notably, the interventions showing the most effectiveness tended to be those that integrated multiple digital tools, utilized a theoretical or conceptual framework for the intervention, and fostered engagement through ‘nudges’ to participants. Most of the interventions employed focus on using digital health as a tool for motivation and encouragement to ensure adherence to a PA plan, and/or sustain healthy behaviors. This approach might explain the positive results indicated in this review. Despite these encouraging findings, the limited geographical coverage of the studies, primarily focusing on Saudi Arabia, Qatar, the United Arab Emirates, Oman, and Egypt, along with the variable sample sizes and research designs employed, indicate the need for larger-scale and more diverse studies. Further studies are necessary to identify the most effective interventions, particularly concerning long-term impact on health outcomes. Through this, digital technologies can be effectively utilized to promote PA and overall health in the Arab region.

## Supplementary Material

Supplementary_material_FINAL_oqaf028

PRISMA-ScR-Fillable-Checklist_10Sept2019_oqaf028

## Data Availability

The authors confirm that the data supporting the findings of this review are available within the article, figure, and tables.

## References

[1] Kardan M, Jung A, Iqbal M et al. Efficacy of digital interventions on physical activity promotion in individuals with noncommunicable diseases: an overview of systematic reviews. *BMC Digital Health* 2024;2:40. 10.1186/s44247-024-00097-6

[2] World Health Organization . Going digital for noncommunicable diseases: the case for action, Geneva, Switzerland: World Health Organization, Accessed Sept 3rd, 2025. https://iris.who.int/handle/10665/378478

[3] Xiong S, Lu H, Peoples N et al. Digital health interventions for non-communicable disease management in primary health care in low-and middle-income countries. *NPJ Digit Med* 2023;6:12. 10.1038/s41746-023-00764-4 36725977 PMC9889958

[4] world Health Organization . Noncommunicable diseases, Geneva, Switzerland: World Health Organization, Accessed Sept 3rd, 2025. https://www.who.int/news-room/fact-sheets/detail/noncommunicable-diseases

[5] Manderson L, Jewett S. Risk, lifestyle and non-communicable diseases of poverty. *Glob Health* 2023;19:13, 13. 10.1186/s12992-023-00914-z PMC9978269 36864476

[6] Mendenhall E, Kohrt BA, Norris SA et al. Non-communicable disease syndemics: poverty, depression, and diabetes among low-income populations. *Lancet (London, England)* 2017;389:951–63. 10.1016/s0140-6736(17)30402-6 28271846 PMC5491333

[7] Chen S, Kuhn M, Prettner K et al. The macroeconomic burden of noncommunicable diseases in the United States: estimates and projections. *PLoS One* 2018;13:e0206702. 10.1371/journal.pone.0206702 30383802 PMC6211719

[8] Lago-Peñas S, Rivera B, Cantarero D et al. The impact of socioeconomic position on non-communicable diseases: what do we know about it? *Perspect Public Health* 2021;141:158–76. 10.1177/1757913920914952 32449467

[9] Murthy S, Kamath P, Godinho MA et al. Digital health innovations for non-communicable disease management during the COVID-19 pandemic: a rapid scoping review. *BMJ Innovations* 2023;9:3–18. 10.1136/bmjinnov-2021-000903

[10] Kazibwe J, Tran PB, Annerstedt KS. The household financial burden of non-communicable diseases in low- and middle-income countries: a systematic review. *Health Res Policy Syst* 2021;19:96. 10.1186/s12961-021-00732-y 34154609 PMC8215836

[11] Mair JL, Salamanca-Sanabria A, Augsburger M et al. Effective behavior change techniques in digital health interventions for the prevention or Management of Noncommunicable Diseases: an umbrella review. *Ann Behav Med* 2023;57:817–35. 10.1093/abm/kaad041 37625030 PMC10498822

[12] Kyaw TL, Ng N, Theocharaki M et al. Cost-effectiveness of digital tools for behavior change interventions among people with chronic diseases: systematic review. *Interact J Med Res* 2023;12:e42396. 10.2196/42396 36795470 PMC9982716

[13] Castro O, Mair JL, Salamanca-Sanabria A et al. Development of "LvL UP 1.0": a smartphone-based, conversational agent-delivered holistic lifestyle intervention for the prevention of non-communicable diseases and common mental disorders. *Front Digit Health* 2023;5:1039171. 10.3389/fdgth.2023.1039171 37234382 PMC10207359

[14] Li WHC, Ho LLK, Cheung AT et al. A general health promotion approach to helping smokers with non-communicable diseases quit smoking: a pilot randomized controlled trial. *Front Public Health* 2022;10:957547. 10.3389/fpubh.2022.957547 36330106 PMC9623171

[15] Boima V, Doku A, Agyekum F et al. Effectiveness of digital health interventions on blood pressure control, lifestyle behaviours and adherence to medication in patients with hypertension in low-income and middle-income countries: a systematic review and meta-analysis of randomised controlled trials. *EClinicalMedicine.* 2024;69:102432. 10.1016/j.eclinm.2024.102432 38333367 PMC10850120

[16] Gold N, Yau A, Rigby B et al. Effectiveness of digital interventions for reducing Behavioral risks of cardiovascular disease in nonclinical adult populations: systematic review of reviews. *J Med Internet Res* 2021;23:e19688. 10.2196/19688 33988126 PMC8164125

[17] Duan Y, Shang B, Liang W et al. Effects of eHealth-based multiple health behavior change interventions on physical activity, healthy diet, and weight in people with noncommunicable diseases: systematic review and meta-analysis. *J Med Internet Res* 2021;23:e23786. 10.2196/23786 33616534 PMC8074786

[18] Mahmood A, Kedia S, Wyant DK et al. Use of mobile health applications for health-promoting behavior among individuals with chronic medical conditions. *Digit Health* 2019; 5:2055207619882181. 10.1177/2055207619882181 31656632 PMC6791047

[19] Morales J, Inupakutika D, Kaghyan S et al. Technology-based health promotion: current state and perspectives in emerging gig economy. *Biocybern Biomed Eng* 2019;39:825–42. 10.1016/j.bbe.2019.07.006 32313347 PMC7169936

[20] Leese C, Abraham K, van de Konijnenburg C et al. The effectiveness and acceptability of digital health interventions as tools to promote physical activity in primary care: an update scoping review. *Prim Health Care Res Dev* 2025;26:e70. 10.1017/s1463423625100339 40815079 PMC12455354

[21] Greenwood SA, Young HML, Briggs J et al. Evaluating the effect of a digital health intervention to enhance physical activity in people with chronic kidney disease (kidney BEAM): a multicentre, randomised controlled trial in the UK. *Lancet Digit Health* 2024;6:e23–32. 10.1016/s2589-7500(23)00204-2 37968170

[22] Wang SCY, Kassavou A. Digital health behavioural interventions to support physical activity and sedentary behaviour in adults after stroke: a systematic literature review with meta-analysis of controlled trials. *Behav Sci (Basel)* 2023;13:62. 10.3390/bs13010062 PMC9855227 36661634

[23] Bi S, Yuan J, Wang Y et al. Effectiveness of digital health interventions in promoting physical activity among college students: systematic review and meta-analysis. *J Med Internet Res* 2024;26:e51714. 10.2196/51714 39566049 PMC11618011

[24] McLaughlin M, Delaney T, Hall A et al. Associations between digital health intervention engagement, physical activity, and sedentary behavior: systematic review and meta-analysis. *J Med Internet Res* 2021;23:e23180. 10.2196/23180 33605897 PMC8011420

[25] Abdul Rahim HF, Sibai A, Khader Y et al. Non-communicable diseases in the Arab world. *Lancet* 2014;383:356–67. 10.1016/S0140-6736(13)62383-1 24452044

[26] Alwan A . Responding to priority health challenges in the Arab world. *Lancet* 2014;383:284–6. 10.1016/S0140-6736(13)62572-6 24452039 PMC7134683

[27] Batniji R, Khatib L, Cammett M et al. Health in the Arab world: a view from within 1: governance and health in the Arab world. *Lancet* 2014;383:343–55. 10.1016/S0140-6736(13)62185-6 24452043 PMC4176927

[28] Mokdad AH, Jaber S, Abdel Aziz MI et al. The state of health in the Arab world, 1990-2010: an analysis of the burden of diseases, injuries, and risk factors. *Lancet* 2014;383:309–20. 10.1016/S0140-6736(13)62189-3 24452042

[29] Rashad H . Health equity in the Arab world: the future we want. *Lancet* 2014;383:286–7. 10.1016/S0140-6736(13)62350-8 24452040

[30] El-Jardali F, Bou-Karroum L, Jabbour M et al. Digital health in fragile states in the Middle East and North Africa (MENA) region: a scoping review of the literature. *PLoS One* 2023;18:e0285226. 10.1371/journal.pone.0285226 37115778 PMC10146476

[31] Cuijpers P, Heim E, Abi Ramia J et al. Effects of a WHO-guided digital health intervention for depression in Syrian refugees in Lebanon: a randomized controlled trial. *PLoS Med* 2022; 19:e1004025. 10.1371/journal.pmed.1004025 35737665 PMC9223343

[32] Al Dweik R, Ajaj R, Kotb R et al. Opportunities and challenges in leveraging digital technology for mental health system strengthening: a systematic review to inform interventions in the United Arab Emirates. *BMC Public Health* 2024;24:2592. 10.1186/s12889-024-19980-y 39334131 PMC11429924

[33] Barakat C, Yousufzai S, Mohammed S et al. Physical activity and sport participation among adolescents from MENA. In: Barakat C, Dghaim R, Al Anouti F, eds. Adolescent Health in the Middle East and North Africa: An Epidemiological Perspective. cham, Switzerland: Springer International Publishing; 2022:51–69, 10.1007/978-3-030-92107-1_4.

[34] Bojorquez I, Romo-Aguilar ML, Ojeda-Revah L et al. Public spaces and physical activity in adults: insights from a mixed-methods study. *Cad Saude Publica* 2021;37:e00028720. 10.1590/0102-311x00028720 33503160

[35] Fletcher GF, Landolfo C, Niebauer J et al. Promoting physical activity and exercise: JACC health promotion series. *J Am Coll Cardiol* 2018;72:1622–39. 10.1016/j.jacc.2018.08.2141 30261965

[36] Jirathananuwat A, Pongpirul K. Promoting physical activity in the workplace: a systematic meta-review. *J Occup Health* 2017;59:385–93. 10.1539/joh.16-0245-RA 28740029 PMC5635147

[37] Nash EA, Critchley JA, Pearson F et al. A systematic review of interventions to promote physical activity in six gulf countries. *PLoS One* 2021;16:e0259058. 10.1371/journal.pone.0259058 34710147 PMC8553078

[38] Rebar AL, Gardner B, Verplanken B. Habit in Exercise Behavior. In Tenenbaum G, Eklund RC. eds., Handbook of Sport Psychology, 2020, 10.1002/9781119568124.ch48

[39] Benjamin K, Donnelly T. Barriers and facilitators influencing the physical activity of Arabic adults: a literature review. *Avicenna.* 2013;2013:8. 10.5339/avi.2013.8

[40] Chaabane S, Chaabna K, Doraiswamy S et al. Barriers and facilitators associated with physical activity in the Middle East and North Africa region: a systematic overview. *Int J Environ Res Public Health* 2021;18:1647. 10.3390/ijerph18041647 33572229 PMC7914747

[41] Daryabeygi-Khotbehsara R, Shariful Islam SM, Dunstan D et al. Smartphone-based interventions to reduce sedentary behavior and promote physical activity using integrated dynamic models: systematic review. *J Med Internet Res* 2021;23:e26315. 10.2196/26315 34515637 PMC8477296

[42] Davis A, Sweigart R, Ellis R. A systematic review of tailored mHealth interventions for physical activity promotion among adults. *Transl Behav Med* 2020;10:1221–32. 10.1093/tbm/ibz190 33044542

[43] Islam MM, Poly TN, Walther BA et al. Use of mobile phone app interventions to promote weight loss: meta-analysis. *JMIR Mhealth Uhealth* 2020;8:e17039. 10.2196/17039 32706724 PMC7407260

[44] Sharara E, Akik C, Ghattas H et al. Physical inactivity, gender and culture in Arab countries: a systematic assessment of the literature. *BMC Public Health* 2018;18:639. 10.1186/s12889-018-5472-z 29776343 PMC5960209

[45] Albabtain AF, AlMulhim DA, Yunus F et al. The role of mobile health in the developing world: a review of current knowledge and future trends. *J Sel Areas Health Inform* 2014;4:10–5

[46] Walsh JC, Groarke JM. Integrating behavioral science with mobile (mHealth) technology to optimize health behavior change interventions. *Eur Psychol* 2019;24:38–48. 10.1027/1016-9040/a000351

[47] El-Zein A, Jabbour S, Tekce B et al. Health and ecological sustainability in the Arab world: a matter of survival. *Lancet (London, England)* 2014;383:458–76. 10.1016/s0140-6736(13)62338-7 24452051 PMC4238938

[48] Horton R . Health in the Arab world: a renewed opportunity. *Lancet* 2014;383:283–4. 10.1016/S0140-6736(13)62703-8 24452038

[49] Bull F, Dvorak J. Tackling chronic disease through increased physical activity in the Arab world and the Middle East: challenge and opportunity. *Br J Sports Med* 2013;47:600–2. 10.1136/bjsports-2012-092109 23624465

[50] Tackling obesity in the Eastern Mediterranean Region . *East Mediterr Health J* 2019;25:142–3. 10.26719/2019.25.2.142 30942479

[51] World Health Organization Eastern Mediterranean Regional Office . Health education and promotion: Physical activity. In: World Health Organization, Cairo, Egypt: World Health Organization Eastern Mediterranean Regional Office, 2017, Accessed May 2, 2017. http://www.emro.who.int/health-education/physical-activity/background.html

[52] Tricco AC, Lillie E, Zarin W et al. PRISMA extension for scoping reviews (PRISMA-ScR): checklist and explanation. *Ann Intern Med* 2018;169:467–73. 10.7326/M18-0850 30178033

[53] Arksey H, O'Malley L. Scoping studies: towards a methodological framework. *Int J Soc Res Methodol* 2005;8:19–32. 10.1080/1364557032000119616

[54] Blair I, Grivna M, Sharif A. The “Arab world” is not a useful concept when addressing challenges to public health, public health education, and research in the Middle East. *Front Public Health* 2014;2:30. 10.3389/fpubh.2014.00030 24783189 PMC3988371

[55] League of Arab States . Member states. Accessed June 1st, 2023. http://www.leagueofarabstates.net/ar/Pages/default.aspx

[56] Ouzzani M, Hammady H, Fedorowicz Z et al. Rayyan—a web and mobile app for systematic reviews. *Syst Rev* 2016;5:210. 10.1186/s13643-016-0384-4 27919275 PMC5139140

[57] Peters MDJ, Godfrey C, McInerney P et al. Chapter 11: Scoping reviews (2020 version). In: Aromataris E, Munn Z, eds. JBI Manual for Evidence Synthesis. 2020, 10.46658/JBIRM-20-01.

[58] Alasfour M, Almarwani M. The effect of innovative smartphone application on adherence to a home-based exercise programs for female older adults with knee osteoarthritis in Saudi Arabia: a randomized controlled trial. *Disabil Rehabil* 2022;44:2420–7. 10.1080/09638288.2020.1836268 33103499

[59] Alnasser A, Kyle J, Aloumi N et al. The Twazon Arabic weight loss app: app-based intervention for Saudi women with obesity. *JMIR Mhealth Uhealth* 2019;7:e10923. 10.2196/10923 31140444 PMC6658271

[60] Alshahrani A, Siddiqui A, Khalil S et al. WhatsApp-based intervention for promoting physical activity among female college students, Saudi Arabia: a randomized controlled trial. *East Mediterr Health J* 2021;27:782–9. 10.26719/emhj.21.012 34486714

[61] Basuodan RM, Bin Sheeha BH, Basoudan NE et al. Tele-Physical Activity Promotion Program among College Students during the COVID-19 Pandemic. Medicina (Kaunas). 2023;59:332. 10.3390/medicina59020332 PMC9960905 36837532

[62] Gmmash A, Alonazi A, Almaddah M et al. Influence of an 8-Week Exercise Program on Physical, Emotional, and Mental Health in Saudi Adolescents: A Pilot Study. Medicina (Kaunas). 2023;59:883. 10.3390/medicina59050883 PMC10223168 37241115

[63] Saquib J, Alhomaidan HT, Al-Mohaimeed A et al. Effect of a WhatsApp walking trial on daily steps among female Saudi Arabian university students. *Int J Health Sci (Qassim)* 2023;17:16–21 36891045 PMC9986880

[64] Ismail T, Al TD. Design and evaluation of a just-in-time adaptive intervention (JITAI) to reduce sedentary behavior at work: experimental study. *JMIR Form Res* 2022;6:e34309. 10.2196/34309 35080498 PMC8943689

[65] Al-Mohannadi AS, Sayegh S, Ibrahim I et al. Effect of a pedometer-based walking challenge on increasing physical activity levels amongst hospital workers. *Arch Public Health* 2019;77:40. 10.1186/s13690-019-0368-7 31572606 PMC6757369

[66] Al-Kuwari MG, Al-Hamdani AY. A comparative study on the uptake and physical activity outcome of the pedometer and smartphone application in Qatar. *Gazzetta Medica Ital Arch per le Sci Mediche* 2021;180:603–6. 10.23736/S0393-3660.21.04616-7

[67] Al-Kuwari MG, Al-Mohannadi AS, El J II et al. Effect of online pedometer program on physical activity in Qatar. *J Sports Med Phys Fitness* 2016;56:275–80. 25503708

[68] Salih , Khidir ED, Stănescu M, Al Sayegh S. *Using mobile technology to evaluate the active lifestyle of adults from campuses in Qatar* 2021;3:404–15

[69] Al-Kuwari MG, Al-Mohannadi AS, Sayegh S. Effectiveness of "step into health" program in Qatar: a pedometer-based longitudinal study. *J Sports Med Phys Fitness* 2017;57:1513–8. 10.23736/s0022-4707.16.06716-5 28004902

[70] Ali HI, Attlee A, Alhebshi S et al. Feasibility study of a newly developed technology-mediated lifestyle intervention for overweight and obese Young adults. *Nutrients.* 2021;13:2547. 10.3390/nu13082547 PMC8399959 34444707

[71] Hasan H, Attlee A, Mohamed JBJ et al. Counting footsteps with a pedometer to improve HMW adiponectin and metabolic syndrome among Young female adults in the United Arab Emirates. *J Obes* 2018;2018:1–9. 10.1155/2018/1597840 PMC6304855 30631594

[72] Al-Ghafri TS, Al-Harthi S, Al-Farsi Y et al. Changes in self-efficacy and social support after an intervention to increase physical activity among adults with type 2 diabetes in Oman: a 12-month follow-up of the MOVEdiabetes trial. *Sultan Qaboos Univ Med J* 2021;21:e42–9. 10.18295/squmj.2021.21.01.006 33777422 PMC7968896

[73] Shaban MM, Sharaa HM, Amer FGM et al. Effect of digital based nursing intervention on knowledge of self-care behaviors and self-efficacy of adult clients with diabetes. *BMC Nurs* 2024;23:130. 10.1186/s12912-024-01787-2 38378505 PMC10877800

[74] Hashim M, Radwan H, Ismail LC et al. Determinants for Mediterranean diet adherence beyond the boundaries: a cross-sectional study from Sharjah, the United Arab Emirates. *J Transl Med* 2024;22:513. 10.1186/s12967-024-05172-0 38807139 PMC11134895

[75] Alodhayani AA, Hassounah MM, Qadri FR et al. Culture-specific observations in a Saudi Arabian digital home health care program: focus group discussions with patients and their caregivers. *J Med Internet Res* 2021;23:e26002. 10.2196/26002 34889740 PMC8701718

[76] Madian AAM, Sayed SH. Effect of interactive digital-based educational intervention on knowledge, misconceptions, and preventive practices of COVID -19 among Damanhour university students. *Egyptian Journal of Health Care* 2021;12:1136–54. 10.21608/ejhc.2021.181876

[77] Hussien NKA, Mokbel RA, Sayed SH et al. Effect of interactive digital-based psychoeducational intervention about COVID-19 on knowledge, fear, and anxiety among secondary school students. *Clinical Schizophrenia & Related Psychoses* 2022;15:1–8. 10.3371/CSRP.KNAR.110321

[78] Huang KY, Kumar M, Cheng S et al. Applying technology to promote sexual and reproductive health and prevent gender based violence for adolescents in low and middle-income countries: digital health strategies synthesis from an umbrella review. *BMC Health Serv Res* 2022;22:1373. 10.1186/s12913-022-08673-0 36401323 PMC9675248

[79] Maaß L, Angoumis K, Freye M et al. Mapping digital public health interventions among existing digital technologies and internet-based interventions to maintain and improve population health in practice: scoping review. *J Med Internet Res* 2024;26:e53927. 10.2196/53927 39018096 PMC11292160

[80] Heerman WJ, Rothman RL, Sanders LM et al. A digital health behavior intervention to prevent childhood obesity: the greenlight plus randomized clinical trial. *Jama.* 2024;332:2068–80. 10.1001/jama.2024.22362 39489149 PMC11533126

[81] Goh CC, Gan XM, Klainin-Yobas P. Effectiveness of digital-based interventions on physical and psychological outcomes among cancer patients: a systematic review and meta-analysis. *Semin Oncol Nurs* 2025;41:151796. 10.1016/j.soncn.2024.151796 39721898

[82] Patel SB, Iqbal FM, Lam K et al. Characterizing Behaviors that influence the implementation of digital-based interventions in health care: systematic review. *J Med Internet Res* 2025;27:e56711. 10.2196/56711 40505130 PMC12203025

[83] Nair US, Howard AM, Piñeiro B et al. Digital health interventions for cancer screening in sexual and gender diverse populations: a scoping review. *Health Promot Pract* 2025;15248399251358719. 10.1177/15248399251358719 40842336

[84] Abu-Ras W, Idris LB, Aboul-Enein BH. Effectiveness of cartoons, comics, and animation-based sexual health promotion and education interventions: a scoping review. *Int J Sex Health* 2025;37:221–39. 10.1080/19317611.2024.2421882 40400565 PMC12091912

[85] Yun J, Shin J, Lee H et al. Characteristics and potential challenges of digital-based interventions for children and Young people: scoping review. *J Med Internet Res* 2023;25:e45465. 10.2196/45465 37058340 PMC10148209

[86] Della Valle C, Gatti C, Bricca A et al. Effects of digital physical activity interventions on muscle mechanical function in community-dwelling older adults: a systematic review and meta-analysis. *Eur Rev Aging Phys Act* 2025;22:14. 10.1186/s11556-025-00380-z 40898014 PMC12403258

[87] Motevalli M, Drenowatz C, Tanous D et al. Guideline-based digital exercise interventions for reducing body weight and fat and promoting physical activity in adults with overweight and obesity: systematic review and meta-analysis. *Interact J Med Res* 2025;14:e73656. 10.2196/73656 40773286 PMC12371298

[88] Zhang X, Fang J, Hao Y et al. Digital behavior change interventions to promote physical activity and reduce sedentary behavior among survivors of breast cancer: systematic review and meta-analysis of randomized controlled trials. *J Med Internet Res* 2025;27:e65278. 10.2196/65278 40537089 PMC12226785

[89] Akgül H, Birtane M, Tonga E. Effects of digital-based physical activity intervention on pain, function and adherence in individuals with knee osteoarthritis: a pilot randomized-controlled trial. *Gait & Posture* 2023;106:S5–6. 10.1016/j.gaitpost.2023.07.011

[90] Al-Shorbaji N, Alhuwail D. Chapter 16—health informatics in the Middle East and North Africa. In: Hovenga E, Grain H, eds. Roadmap to Successful Digital Health Ecosystems. Cambridge, MA: Academic Press; 2022: 375–97, 10.1016/B978-0-12-823413-6.00029-X.

[91] Benson J, Brand T, Christianson L et al. Localisation of digital health tools used by displaced populations in low and middle-income settings: a scoping review and critical analysis of the participation revolution. *Confl Health* 2023;17:20. 10.1186/s13031-023-00518-9 37061703 PMC10105546

[92] Holeman I, Cookson TP, Pagliari C. Digital technology for health sector governance in low and middle income countries: a scoping review. *J Glob Health* 2016;6:020408. 10.7189/jogh.06.020408 27648255 PMC5017033

[93] Weirauch V, Soehnchen C, Burmann A et al. Methods, indicators, and end-user involvement in the evaluation of digital health interventions for the public: scoping review. *J Med Internet Res* 2024;26:e55714. 10.2196/55714 38819891 PMC11179021

[94] Aboye GT, Vande Walle M, Simegn GL et al. Current evidence on the use of mHealth approaches in sub-Saharan Africa: a scoping review. *Health Policy and Technology* 2023;12, 12:100806. 10.1016/j.hlpt.2023.100806

