# Supplementary material for: Digital-based physical activity interventions implemented across the League of Arab States: a scoping review
Source: Oxf Open Digit Health. 2025 Nov 3;3:oqaf028. doi: 10.1093/oodh/oqaf028 (PMC12622223; doi:10.1093/oodh/oqaf028)
Supplement: Supplementary_material_FINAL_oqaf028 [file supplementary_material_final_oqaf028.docx]

**Supplemental material. Scopus database**

“online” [All Fields]; OR “app” [All Fields]; OR “digital” [All Fields]; OR “computer” [All Fields]; OR “technology” [All Fields] OR “internet” [All Fields] OR “web-based” [All Fields] OR “social media” [All Fields] OR “eHealth” [All Fields] OR “mHealth” [All Fields] OR “smartphone” [All Fields]

AND “Physical Activity” [All Fields]; OR “Exercise” [All Fields]

AND “Intervention” [All Fields]; OR “Program” [All Fields]

AND “Algeria”; “Egypt”; “Bahrain”; “Comoros”; “Djibouti”; “Iraq”; “Jordan”; “Saudi Arabia”; “Kuwait”; “Lebanon”; “Libya”; “Mauritania”; “Morocco”; “Oman”; “Occupied Palestinian Territories”; “Qatar”; “Yemen”; “Somalia”; “Sudan”; “Syria”; “Tunisia”; “the United Arab Emirates” [All Fields]
